# Effects of acute and chronic exercise on the osmotic stability of erythrocyte membrane of competitive swimmers

**DOI:** 10.1371/journal.pone.0171318

**Published:** 2017-02-02

**Authors:** Lara Ferreira Paraiso, Ana Flávia Mayrink Gonçalves-e-Oliveira, Lucas Moreira Cunha, Omar Pereira de Almeida Neto, Adriana Garcia Pacheco, Karinne Beatriz Gonçalves Araújo, Mário da Silva Garrote-Filho, Morun Bernardino Neto, Nilson Penha-Silva

**Affiliations:** 1 Institute of Genetics and Biochemistry, Federal University of Uberlândia, Uberlândia, Minas Gerais, Brazil; 2 Department of Health Sciences, Federal University of Goiás, Jataí, Goiás, Brazil; 3 Department of Basic and Environmental Sciences, University of São Paulo, Lorena, São Paulo, Brazil; Newcastle University, AUSTRALIA

## Abstract

This study aimed to evaluate the influence of acute and chronic exercise on erythrocyte membrane stability and various blood indices in a population consisting of five national-level male swimmers, over 18 weeks of training. The evaluations were made at the beginning and end of the 1st, 7th, 13th and 18th weeks, when volume and training intensity have changed. The effects manifested at the beginning of those weeks were considered due to chronic adaptations, while the effects observed at the end of the weeks were considered due to acute manifestations of the exercise load of that week. Acute changes resulting from the exercise comprised increases in creatine kinase activity (CK) and leukocyte count (Leu), and decrease in hematocrit (Ht) and mean corpuscular volume (MCV), at the end of the first week; increase in the activities of CK and lactate dehydrogenase (LDH), in the uric acid (UA) concentration and Leu count, at the end of the seventh week; increases in CK and LDH activities and in the mean corpuscular hemoglobin concentration (MCHC), at the end of the 13th week; and decrease in the value of the osmotic stability index 1/H_50_ and increases in the CK activity and platelets (Plt) count, at the end of the 18th week. Chronic changes due to training comprised increase in the values of 1/H_50_, CK, LDH, high-density lipoprotein cholesterol (HDL-C), low-density lipoprotein cholesterol (LDL-C), serum iron (Fe), MCV and Plt. Although acute training has resulted in decrease in the osmotic stability of erythrocytes, possibly associated with exacerbation of the oxidative processes during intense exercise, chronic training over 18 weeks resulted in increased osmotic stability of erythrocytes, possibly by modulation in the membrane cholesterol content by low and high density lipoproteins.

## Introduction

Physical exercise increases oxygen (O_2_) consumption by the body, mainly in the muscle tissues [1, 2]. In the case of athletes, a good oxygen uptake is extremely important for tolerance to severe exercise [3, 4]. Red blood cells (RBC) should be efficient in O_2_ uptake and delivery to meet the high demand of tissues [5]. The structure of the membranes of these cells plays an important role in maintaining its functionality; an imbalance in the physicochemical properties of the membrane can make the cell to become dysfunctional, hindering tissue oxygenation [6, 7].

It is known that in athletes regular training is able to make changes and adaptations in the blood rheology, including changes in hematological and biochemical parameters [8–12]. These changes can affect membrane properties, such as its stability. The stability can be defined as the membrane's ability to resist lysis against many harmful agents. Many factors that affect the stability of the membrane are well known; among them are diet, age, temperature, use of drugs and some physiological and pathological conditions [13–17].

The osmotic fragility test is a valuable tool to assess the sensitivity of the red cells to osmotic changes in the environment. It is widely used to elucidate the mechanisms affecting the properties of the membrane of erythrocytes [18–20]. The analysis of membrane properties using erythrocytes as a model has the advantages of simplicity of the cell, and the easiness in monitoring their lysis by spectrophotometric measurement of the amount of hemoglobin released into the medium [16, 21, 22].

The number of studies involving exercise and membrane stability is scarce [4, 17, 23, 24]. Little is known about the role that exercise plays in the erythrocyte membrane. It was in this context that the present study aimed to evaluate the acute and chronic changes in the erythrocyte membrane stability and in hematologic and biochemical indices of professional swimmers engaged in an 18-week pre-competition training program.

## Materials and methods

### Population

This study was approved by the Committee of Ethics and Human Research of the Federal University of Uberlândia (337/11). Each volunteer involved in the research signed a free and informed consent term. The study included five (5) national level male swimmers (average age of 24±2.3 years; average body mass index of 24.5±1.6 kg/m^2^). All participants were healthy and did not have any injury or damage to health that could influence the practice of physical exercise. They were non-smokers and non-consumers of alcohol, abuse drugs and long-term prescription medications.

### Protocols of training and blood collection

All athletes were studied since the beginning of the training period that preceded the main competition from which they were going to participate, totaling 18 weeks of training. The evaluations were made at the beginning and end of the 1st, 7th, 13th and 18th weeks, when volume and training intensity have changed (Table 1). The effects manifested the beginning of those weeks were considered due to chronic adaptations, while the effects observed at the end of the week were considered due to acute manifestations of the exercise load of that week.

**Table 1:** Characterization of training sessions.

Week	Volume (km/week)*	Intensity (MIC)**
1	18	50–70%
7	50	72–82%
13	40	85–90%
18	30	88–100%

* The volume was represented by the distance in kilometers that the athlete swam weekly

**MIC: Maximum Individual Capacity. The intensity was related to the swimming time in relation to individual values obtained in previous competitions for each athlete. The closer each athlete was in relation to his maximum swimming time, the greater the intensity of his training.

Blood samples were collected before the athletes perform any physical effort by venipuncture, always in evacuated tubes (Vacutainer; Becton Dickinson, Juiz de Fora, Brazil) containing EDTA as an anticoagulant for determination of erythrogram and evaluation of the erythrocyte membrane stability, and in tubes without anticoagulant, for biochemical assays.

### Determination of hematologic and biochemical variables

The erythrogram (automated Cell-Dyn 3700, Abbott Diagnostics, Abbott Park, IL, USA) and biochemical tests (automated analyzer Architect C 8000, Abbott Diagnostics) were carried out in the Clinical Analysis Laboratory of the Clinical Hospital of the Federal University of Uberlândia. The osmotic fragility test used to evaluate the stability of erythrocytes was conducted at the Laboratory of Biophysical Chemistry of the Federal University of Uberlândia.

The analyzed hematological variables and their reference values were: erythrocytes (RBC), 4.3–5.7 millions/mm^3^; hemoglobin (Hb), 13.0–17.5%; hematocrit (Ht), 39–50%; mean corpuscular volume (MCV), 81.0–95.1 fL; mean corpuscular hemoglobin (MCH), 26–34 pg; mean corpuscular hemoglobin concentration (MCHC), 31–36 g/dL; red-cell distribution width (RDW), 12–15%; leucocytes (Leu), 3.5–10.5 mil/mm^3^; platelets (Plt), 150–450 mil/mm^3^; uric acid (UA), 3.5–7.2 mg/dL; creatine kinase (CK), 30–200 U/L; lactate dehydrogenase (LDH), 100–190 U/L; serum iron (Fe), 50–160 μg/dL; total cholesterol (t-C), <170 (optimum) and ≥240 mg/dL(high); high density lipoprotein cholesterol (HDL-C), ≥40 mg/dL (ideal); low density lipoprotein cholesterol (LDL-C), <130 (optimum) and≥160 mg/Dl (high); very low density lipoprotein cholesterol (VLDL-C), <40 mg/dL; and triglycerides (TGC), <150 (optimum) and ≥200 mg/dL (high).

### Evaluation of the osmotic stability of the erythrocyte membrane

The NaCl solutions (Labsynth, Diadema, SP, Brazil) were prepared using high purity reagent (99%) in ultrapure water (Millipore Corporation, São Paulo, Brazil). The mass measurements were made using analytical balance (Shimadzu, AW220 model, Japan). Volume measurements were made using automatic pipettes (Labsystems, Helsinki, Finland). Incubations were performed in a thermostatic bath (model 184 MA; Marconi, Piracicaba, Brazil). The absorbance readings were made in a digital spectrophotometer (model UV-1650; Shimadzu, Tokyo, Japan). Centrifugations were performed in a refrigerated centrifuge (model II CF15RX; Hitachi Koki, Hitachinaka, Japan).

The osmotic fragility test was conducted in a duplicate series of microtubes (Eppendorf, Hamburg, Germany) containing 1.5 mL of NaCl at concentrations ranging from 0.1 a 0.9 g/dL. Initially, the tubes were pre-incubated for 10 minutes at 37°C in the thermostated bath. After pre-incubation, 10 μL of blood were added to each microtube. After homogenization, the microtubes were incubated for 30 min at 37°C. Then the microtubes were centrifuged at 1600 x *g* for 10 minutes at 37°C and their supernatants were subjected to absorbance readings at 540 nm.

**Fig 1:**
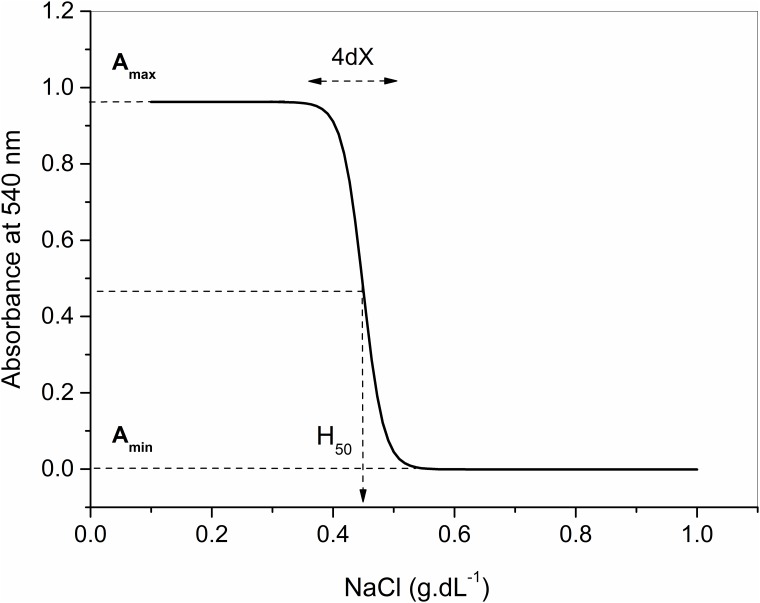
Typical curve of erythrocytes lysis curve by decrease in the NaCl concentration. Data were adjusted by sigmoidal regression. H_50_ is the salt concentration needed to promote 50% hemolysis and 4dX is the variation in salt concentration needed to promote 100% hemolysis associated with the transition from the minimum residual value (A_min_) to the maximum value of absorbance (A_max_). As dX and H_50_ have direct and reverse associations with the osmotic stability of erythrocytes, values of 1/H_50_ and dX were always used in this study in order to deal just with variables directly proportional to osmotic stability.

The graphs of absorbance at 540 nm (A_540_) as a function of the NaCl concentration (X) were fitted to sigmoidal regression lines (Fig 1) according with the Boltzmann equation:

$${\text{A}}_{\text{540}}=\;\frac{{\text{A}}_{\text{min}}-{\text{A}}_{\text{max}}}{{1+\text{e}}^{{\text{(X-H}}_{\text{50}}\text{)/dX}}}{\text{+A}}_{\text{max}},$$ (1)

where **A**_**min**_ (absorbance units) and **A**_**max**_(absorbance units) represent respectively the mean values of **A**_**540**_ at the minimum and maximum plateaus, **H**_**50**_ (g/dL NaCl) is the NaCl concentration capable of promoting 50% hemolysis, and **dX** (g/dL NaCl) is the variation in the salt concentration that is responsible for the complete hemolysis transition. The parameters dX and 1/H_50_ were used to evaluate the osmotic stability of the erythrocyte membrane, since they present same direction associations [17, 25].

### Statistical analyzes

All statistical tests were done using the software Bioestat 5.3 (Mamirauá, Belém, PA, Brazil).

The D'Agostino-Pearson test was used to investigate the existence of normality in the obtained data. The results that were obtained for some variables were not normally distributed. For this reason, comparisons were performed using a non-parametric test for small samples, Wilcoxon Signed Rank Test, which use median values to make comparisons by ranks.

The Wilcoxon Signed Rank Test was used to compare pre-training with post-training times in each of the four collections and to compare the pre-training of first week with the pre-training the 18th week, which allows to investigate the chronic effect of exercise on the analyzed variables. The significance value used in this test was p≤0.063.

## Results

Fig 1 shows a typical curve of dependency of the amount of released hemoglobin, given the absorbance at 540 nm, in function of the NaCl concentration in the medium.

**Fig 2:**
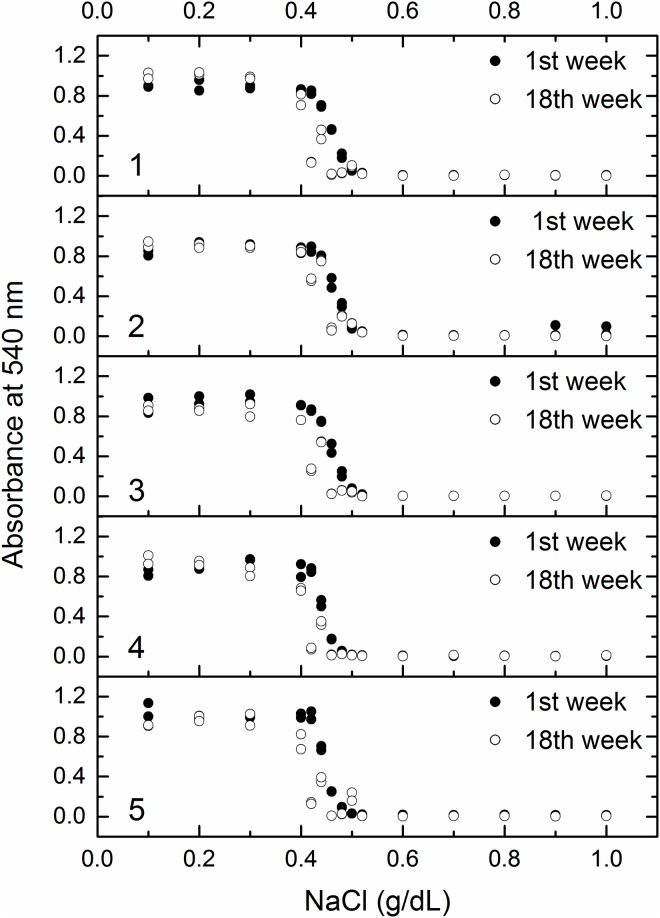
Osmotic fragility curves of red blood cells in the pre-training of the first and 18th weeks of the training program for each of the five volunteers of the study.

Fig 2 shows the variations in the osmotic stability curve in the pre-training of the first week and 18th weeks of the training program for each of the five volunteers of the study.

Table 1 shows the characterization of the training sessions.

Table 2 shows the median and interquartile deviations (IQR) obtained for each of the variables at the beginning and end of each of the four weeks considered in the study, as well as the results of comparisons made by using the Wilcoxon Signed Rank Test. As previously mentioned, the effects manifested after the end of each week of training were considered to be due to acute manifestations of that week exercise load. At the end of the 1st week the parameters that have undergone significant acute changes were CK and Leu, which increased, and Ht and MCV, which reduced. At the end of the 7th week, the values of parameters CK, LDH, UA and Leu increased significantly. At the end of the 13th week there were significant increases in A_max_, CK, MCH and MCHC. At the end of the 18th week there were significant increases in CK and Plt, and a significant decrease in 1/H_50_.

**Table 2:** Comparison between the values of variables before (pre-training) and after (post-training) each training session.

Variables		Weeks of regular training			
		(Number of subjects)			
		1st	7th	13th	18th
		(5)	(5)	(5)	(5)
		Median (IQR)	Median (IQR)	Median (IQR)	Median (IQR)
A_max_ (AU)	Pre-training	0.89 (0.05)	0.96 (0.07)	0.90 (0.01)	0.91 (0.05)
	Post-training	0.88 (0.04)	0.96 (0.13)	1.01 (0.06) ^c^	0.90 (0.03)
A_min_ (AU)	Pre-training	0.00 (0.01)	0.00 (0.00)	0.00 (0.00)	0.02 (0.02)
	Post-training	0.00 (0.00)	0.01 (0.00)	0.00 (0.00)	0.01 (0.00)
1/H_50_ ((g/dL)^-1^ NaCl)	Pre-training	2.17 (0.06)	2.26 (0.04)	2.25 (0.03)	2.44 (0.1) ^a^
	Post-training	2.19 (0.09)	2.29 (0.04)	2.31 (0.03)	2.25 (0.05) ^d^
dX (g/dL NaCl)	Pre-training	0.01 (0.00)	0.01 (0.00)	0.01 (0.00)	0.01 (0.01)
	Post-training	0.01 (0.00)	0.01 (0.00)	0.01 (0.00)	0.01 (0.00)
UA (mg/dL)	Pre-training	4.46 (0.68)	5.42 (0.87)	5.04 (0.14)	5.4 (0.2)
	Post-training	4.58 (0.59)	7.43 (0.83)^b^	5.36 (0.15)	5.7 (0.9)
RBC (10^6^/mm^3^)	Pre-training	5.34 (0.43)	5.45 (0.36)	5.5 (0.28)	5.42 (0.81)
	Post-training	5.19 (0.43)	5.19 (0.47)	5.38 (0.13)	5.03 (0.16)
Hb (g%)	Pre-training	15.8 (0.4)	16.1 (0.5)	15.7 (0.6)	15.6 (0.6)
	Post-training	15.6 (0.7)	15.3 (0.6)	15.3 (1.2)	15.5 (0.2)
Ht (%)	Pre-training	48.7 (2.3)	49.7 (2.1)	49 (1.2)	48.1 (4.1)
	Post-training	47 (1.8) ^a^	47 (3.4)	46.4 (2.6)	45.2 (0.4)
MCV (fL)	Pre-training	91.7 (3.4)	91.5 (2.5)	89.1 (3.1)	90.6 (4.2) ^a^
	Post-training	91 (1.8) ^a^	91.4 (2.3)	86.9 (2.5)	90.3 (2.3)
MCH (pg)	Pre-training	29.7 (0.7)	29.8 (0.9)	28.4 (1.4)	30.6 (1.4)
	Post-training	29.9 (1)	29.8 (0.8)	28.7 (1.4) ^c^	31.1 (1.1)
MCHC (g/dL)	Pre-training	32.4 (0.1)	32.3 (0.5)	31.8 (0.4)	33.2 (0.7)
	Post-training	32.9 (0)	32.3 (0.3)	32.7 (0.7) ^c^	34.4 (0.7)
RDW (%)	Pre-training	14.8 (0.2)	15.5 (1.3)	12 (1)	14.6 (0.6)
	Post-training	14.6 (0.9)	15.4 (0.9)	11 (1)	15.1 (0.4)
Leu (10^3^/mm^3^)	Pre-training	6.4 (1.4)	5.9 (1.4)	6 (2.2)	6 (0.4)
	Post-training	8.4 (1.7) ^a^	10.1 (4.4)^b^	8.8 (2.8)	8.6 (4.4)
Plt (10^3^/mm^3^)	Pre-training	232 (27)	236 (17)	252 (41)	256 (9) ^a^
	Post-training	247 (31)	249 (36)	262 (25)	288 (25) ^c^
Fe (μg/dL)	Pre-training	58 (0.8)	89.6 (11.8)	88.9 (41.3)	106.5 (32.8) ^a^
	Post-training	52.6 (9.2)	76.6 (24.6)	87.4 (24.9)	99.7 (35.4)
CK (U/L)	Pre-training	183 (93)	183 (108)	180 (61)	360 (49) ^a^
	Post-training	230 (97) ^a^	389 (68)^b^	301 (104) ^c^	496 (69) ^c^
LDH (U/L)	Pre-training	144 (26)	180 (15)	211 (17)	255 (76) ^a^
	Post-training	164 (20)	241 (22)^b^	201 (29)	249 (6)
t-C (mg/dL)	Pre-training	163 (22)	222 (36)	190 (56)	198 (32)
	Post-training	143 (28)	210 (52)	195 (9)	203 (26)
TGC (mg/dL)	Pre-training	127 (71)	116 (20)	117 (6)	107 (49)
	Post-training	163 (67)	100 (14)	98 (28)	182 (25)
HDL-C (mg/dL)	Pre-training	40.5 (2.4)	59.5 (11.3)	60.3 (13.4)	58.4 (4.2) ^a^
	Post-training	40 (2.7)	58.7 (5.1)	57.5 (16.6)	58.5 (4.2)
VLDL-C (mg/dL)	Pre-training	23 (24.4)	20.6 (3.4)	23.4 (1.8)	21.4 (9.8)
	Post-training	28 (13.6)	20.8 (3.8)	19.6 (5.6)	36.4 (5)
LDL-C (mg/dL)	Pre-training	76.7 (12.3)	142.7 (43.4)	102 (38.1)	119.6 (6.5) ^a^
	Post-training	82.1 (33.7)	137.1 (38.1)	101.5 (30.6)	104.5 (29.9)

^a^p<0.063 indicates a statistically significant difference in relation to pre-training of the first collect;

^b^p<0.063 indicates a statistically significant difference in relation to pre-training of the second collect;

^c^p<0.063 indicates a statistically significant difference in relation to pre-training of the third collect;

^d^p<0.063 indicates a statistically significant difference in relation to pre-training of the fourth collect.

As also previously mentioned, the effects that have persisted at the beginning of each week were considered to be due to chronic adaptations of the exercise program. Since 1/H_50_ increased in the last week of training in relation to the first week (Table 2), this should mean that the chronic training increased significantly the stability of the RBC membrane. There were also increases in CK, LDH, HDL-C, LDL-C, Fe and Plt, and a significant decrease in the hematological index MCV at the end of the training program (Table 2).

## Discussion

The major finding of our research was that the training, both acute and chronic, has affected the osmotic stability of erythrocytes, as well as the values of various other parameters studied here. The body of the athletes was affected in some way in every training session, even in the first training, which was a light training, in which neither the intensity nor the volume of exercise were high.

The reduction of 1/H_50_ after the acute training with the highest intensity among all (18th week) means that there was a decrease in osmotic stability after this training session. This finding is in agreement with the results found by Paraiso et al., who reported a significant reduction in the value of 1/H_50_ when participants performed a high intensity training [17].

In addition to this decline in 1/H_50_, at the end of 18th week, the serum levels of CK and the Plt counts also have changed, however they had suffered significant increases. In fact, increases in the values of these parameters have often been described in high-intensity exercise [8, 26]. Elevation in platelet count is indicative of oxidative stress and inflammation as a result of performing physical exercise [27–31].

Although this study has not assessed oxidative markers, the relation between exercise and production of reactive oxygen and nitrogenspecies (RONS) has been reported by several authors [4, 23, 32–38]. Overproduction of RONS is a result of a variety of stressors such as exposure to pollutants [39], excessive intake of energetic nutrients [40], and physical exercise [41]. Any situation that induces an abrupt increase in oxygen consumption may result in an acute state of oxidative stress [34]. The production of RONS depends on the type (aerobic or anaerobic), intensity [29, 42–44] and duration of the physical exercise [34, 45–47].

Ciancarelli-Tozzi et al. suggested in their study that oxidative stress associated with strenuous exercise produces activation of platelets and causes a reduction in membrane fluidity of these cells. Platelet activation process is mediated by the production of oxidized LDL molecules that inhibit nitric oxide production, an important component of the activation and inhibitor of platelet aggregation [29].

Oxidative stress is also an important factor associated with the reduction in membrane stability, since it is capable of destabilizing the plasma membrane [17], increasing its permeability [48] and reducing its fluidity [29, 49, 50]. It is possible that the decrease in the osmotic stability of erythrocytes observed *in vitro* after intense training in this study is directly related to oxidation processes promoted by this specific type of training, which would have destabilized *in vivo* the membrane of those cells.

Oppositely to the acute effects, the chronic effects of training, measured at the beginning of each week considered in the present study, comprised an increase in 1/H_50_ and consequently in the membrane stability over time. Few studies have evaluated the chronic effect of exercise on the properties of the RBC membrane [51, 52].

Some studies have used athletes in this type of research [1, 3, 4, 24], but no studies have evaluated the cumulative effect of training athletes in the osmotic membrane stability. The studies found did not evaluate athletes before and after the training period, but just compared the individuals already in training with sedentary individuals in relation to the erythrocyte stability [1, 3]. A key differentiator of this study was the use of the athlete himself, before being submitted to the training period, as a control, to investigate the influence of exercise, what was possible because the assessed athletes were coming from a vacation period in which they do not trained.

Kamada et al. evaluated the membrane fluidity of red cells from runner athletes, cyclists and sedentary individuals. The athletes presented higher membrane fluidity compared to sedentary individuals. The authors attributed this increased membrane fluidity in athletes to the occurrence of changes in the lipid composition of the membrane in order to improve the efficiency of oxygen delivery by microcirculation, helping athletes to tolerate the stress of severe exercise [3]. Cazzola et al. also found a greater fluidity of erythrocyte membrane in soccer players when compared to sedentary individuals. They associated the greater fluidity to better antioxidant status and also the characteristics of hematological indices of footballers. The volunteers in this group had higher values of MCV, MCHC and Ht, compared with the group of sedentary individuals [1].

In this study, some hematologic indices suffered changes from the first to the last week of training. The results suggest that training has promoted a change in the characteristics of red blood cells, with a predominance of smaller erythrocytes at the 18th week, since there was a significant decrease in MCV. This may be due to the development of a nutritional deficiency frame during the training period, since selective nutritional deficiencies associated with training can affect erythropoiesis. Indeed, Shiraki et al. reported a decrease in the number of erythrocytes over a period of 21 days of training in active individuals with a low protein diet when compared to subjects with medium and high intake of protein. Despite the reduction in the number of red blood cells observed in these subjects, there was a higher production of reticulocytes during the training period, especially in the group with higher protein intake. These changes were reflected in the osmotic fragility, since the regular training was associated with an increase in the osmotic stability of erythrocytes. In this study, the authors were able to conclude that exercise activates erythropoiesis, but the red blood cell production is closely related to nutritional status, especially with the protein status of the individual [52].

Furthermore, the decrease in osmotic fragility is also related to acceleration of the turnover [53] and increase in the number of young erythrocytes, since they are more resistant to lysis in hypotonic saline solutions [54, 55]. Unfortunately the present study did not assess the nutritional status of individuals, but only the levels of serum iron, which remained within the reference range. The significant increase in this parameter at the end of the 18 training weeks may be an indication of increased cell turnover as an adaptive response to exercise, because iron is essential for the RBC production and, consequently, to the transport O_2_ and CO_2_ through the body [56]. The increase in cell turnover due to chronic training is a process shown by many authors as being an adaptive response of the body to remove old cells, which exhibit alterations in their structure and rheology, for young cells, which are more stable and more efficient in performing their transport functions [57].

Another factor that is also involved in the process of acceleration of cell turnover caused by exercise is the increased intravascular lysis of erythrocytes during physical effort [58, 59]. Spodaryk stated that the training of swimmers stimulates erythropoiesis and that old cells are eliminated faster due to increased intravascular hemolysis that occurs during the course of exercising [60].

Over the weeks of training there were significant elevations in serum levels of HDL-C and LDL-C, but still staying within the reference range. These elevations may be related to modulation of the membrane fluidity to enhance its functionality, since the content of cholesterol in erythrocyte membrane reflects the blood cholesterol levels of high and low density lipoproteins [61, 62]. The increase in the cholesterol content until a critical level allows the membrane to become more stable and have the critical fluidity which is necessary to perform its functions. Beyond this critical level, the increase in the cholesterol content leads to decrease in membrane fluidity and impairment of its functions [63]. HDL plays a major role throughout the body by removing excess cholesterol from a membrane, so that it can congregategreater stability with the critical flow necessary to perform their functions. The increase in HDL-C levels shown in this paper indicates the occurrence of an increase in the efficiency of the mechanisms of excess cholesterol removal from the membranes of extra-hepatic tissues and cells to ensure the critical membrane fluidity and promote the so-called reverse transport of cholesterol to the liver.

Despite the small sample size, a common limitation in studies involving high-level athletes, significant results were found in this study. Although acute exercise have led to the decrease in osmotic stability of erythrocytes, chronic training for 18 weeks has led to an increase in the osmotic stability of erythrocytes, possibly by the modulation of the membrane cholesterol content by the low and high density lipoproteins.

## Supporting information

S1 File Database. Results obtained through the blood analysis of each study volunteer.
(XLSX)
